# Ceftriaxone Resistance in the Surgical Adult Intensive Care Unit at Muhimbili National Hospital, Dar es Salaam, Tanzania

**DOI:** 10.24248/eahrj.v9i2.860

**Published:** 2025-12-24

**Authors:** Gibonce Mwakisambwe, Edwin Lugazia

**Affiliations:** a Department of Anesthesia and Critical Care, Mbeya Zonal Referral Hospital, Tanzania; b Department of Anesthesia and Critical Care, School of Medicine, University of Dar es Salaam-Mbeya College of Health and Allied Sciences, Tanzania; c Department of Anesthesia and Critical Care, School of Clinical Medicine, Muhimbili University of Health and Allied Sciences, Tanzania

## Abstract

**Background:**

The resistance to first line agents, namely third generation cephalosporins, specifically ceftriaxone, has been clinically observed in a number of patients at Muhimbili National Hospital (MNH) medical wards. However, there has been no any study from the intensive care units with regards to resistance to ceftriaxone per se.

**Objective:**

The objective of our study was to audit the use of ceftriaxone with STG adherence as the first line antibacterial agent on the prophylaxis and treatment of bacterial infections among patients admitted in the surgical Adult Intensive Care Unit (AICU) at MNH, Dar es Salaam, Tanzania.

**Methods:**

This was a prospective descriptive cross-sectional clinical audit study, done from January, 2021 to December, 2021. Results were analyzed using SPSS 23.

**Results:**

During one year of the study period, a total of 735 patients were admitted in the surgical MNH-AICU with different clinical diagnosis. A total of 731 (99.50%) were included in the final analysis, with only 75 (10.26%) having culture and sensitivity performed on them. Among all isolates, *Klebsiella pneumoniae* 34 (45%) was the most predominant bacteria. *Klebsiella pneumonia*, *Acinetobacter spp* and *Escherichia coli* were 100% resistant to ceftriaxone (CRO) while five isolates of *K. pneumoniae* were resistant to all tested antibiotics including Meropenem. The STG adherence was only in 26 (4.30%) patients out of 731 patients studied with regards to ceftriaxone prescription.

**Conclusion:**

In this study, the majority of pathogens were due to multi-drug resistant (MDR) bacteria. The STG adherence was very low with regard to the use of ceftriaxone (4.30%). The resistance to Ceftriaxone was 100%; thus, Ceftriaxone should no longer be used as the first line for prophylaxis and treatment of bacterial infections in the surgical MNH-AICU and similar settings. Crucially, the establishment of dedicated Antimicrobial Stewardship Program (ASP) teams, along with rigorous education and strict adherence to defined guidelines, is of paramount importance to counteract and effectively reduce the observed antimicrobial resistance (AMR) burden.

## BACKGROUND

Massive and inappropriate use of ceftriaxone has led to an enormous rise in resistance to commonly used third-generation cephalosporins, including ceftriaxone itself, as per study performed at Muhimbili National Hospital (MNH).^1^ The ICU at MNH admits patients from all disciplines and a range of ages from different parts of the country. The mixed nature of our patients gives a wide spectrum of responsible pathogens, which makes the management of sepsis complex in regard to antibiotic resistance. Previous studies in Tanzania have reported that approximately 7% and 20% of neonatal mortalities in tertiary hospitals are associated with infectious diseases.^2,3^ By not knowing the existing problem, we cannot provide exact antibiotics with respect to causative organisms without culture and sensitivities being done to all septic patients.

The main purpose of the antimicrobial surveillance (AMS) includes making use of the antimicrobial agent in appropriate patient, appropriate indication, proper drug, proper administration of the dose, route, duration of treatment, use of antibiotics in appropriate combination, and its effective cost.^4^ That has been shown to encourage an effective antibiotic prescription to improve the clinical outcomes within the intensive care unit (ICU) settings for the critically ill patients.^5^

In resource-limited settings such as Tanzania, delayed diagnosis of sepsis is the major factor contributing to the observed high mortality associated with sepsis in intensive care units. Criteria in the past have included systemic inflammatory response syndrome (SIRS). In HIC, SIRS has helped in the identification; however, implementation of SIRS in resource-limited settings has been difficult due to laboratory requirements. To address this issue, the newly published sepsis guidelines have made efforts to use a simple parameter, bedside-based screening tool for sepsis.^6^

At Kilimanjaro Christian Medical Centre (KCMC), one study performed in the neonatal unit, mortalities due to sepsis has been reported to be almost 20%; due to multidrug resistance (MDR) strains,^2^ with higher figures than those reported in HICs, accounting for the observed higher mortality. Timely blood culture and prompt administration of antibiotics have been shown to be associated with lower mortality.^7^ Additionally, identification allows de-escalation of antibiotic therapy, which is associated with lower mortality.

A recent study from a Ugandan hospitals showed that resistance to ceftriaxone has increased up to 78.6%.^8^ Successful management of sepsis relies mainly on the correct identification of the condition and prompt treatment.^9^ In sub-Saharan Africa (SSA), the expected burden of sepsis can be estimated by looking at HIV/AIDS outcomes. There are currently 36.7 million people infected with HIV globally, of whom 19 million are in SSA, representing 51% of the total HIV global burden.^10^ Sepsis is a complex syndrome that can arise from any infection and is a significant cause of mortality and morbidity worldwide.^11^

Looking specifically at surgical MNH-AICUs, sepsis is the most common cause of admission, with mortality ranging from 20% to 50%. The global in-hospital mortality of sepsis is 17% and increases to 27% in cases of severe sepsis. In addition to lost life, there is a significant financial burden, with an estimated $16.7 billion spent annually with a projected 1.5% increase per annum.^12^

We observed the trends towards high resistance on ceftriaxone in the surgical MNH-AICUs, hence the need to conduct the study in order to understand the knowledge on the correct prescription as per Tanzanian STG.

This prospective descriptive cross-sectional study audited the use of ceftriaxone as the first-line antibacterial agent for the prophylaxis and treatment of bacterial infections among patients admitted to the surgical MNH-AICUs.

## METHODOLOGY

### Study Design and Area

This was a prospective descriptive cross-sectional clinical audit on the use of ceftriaxone as the first-line antibacterial agent for the prophylaxis and treatment of bacterial infections among patients admitted to the surgical MNH-AICUs. Muhimbili is a national consultant hospital that serves patients from all over the country, admitting at least 300 critically ill patients per year, with 20–40 patients per month, in both the pediatric and adult ICUs. The ICU is divided into different subunits: surgical, medical, and pediatric units. Primary care providers vary in these ICUs with regard to those given the sole mandate to be the primary care providers, including anesthesiologists, intensivists, pulmonologists, registrars, residents and surgeons, and their numbers average 4, 2, 4, 4, 12 and 6, respectively; they are also the main prescribers for theMNH-ICUs.

### Study Population

All patients admitted to the surgical MNH-AICU from all disciplines were included in the audit as they were all candidates to receive ceftriaxone.

### Sample Size Estimation

Cochran's formula was used, with the minimum estimated sample size of 384 patients.

### Data Collection

Admission books and the Jeeva system were used to obtain the names and registration numbers of the patients; thereafter, the patient's medical record files were tracked from the medical record department and the MNH main laboratory. Permission to access patient information from the Jeeva System was sought from the MNH-Main laboratory authorities. The correct use of ceftriaxone was defined by using the STG adherence for the specific diagnosis. Liquid medium jars were used for sample collection in aseptic technique, for both, aerobic and anaerobic cultures.

All appropriate investigations were recorded. The primary outcomes were the common microbes associated with MDR in the surgical MNH-AICU and the prevalence of resistance to ceftriaxone. Data were collected using the international adopted clinical auditing data sheet/tool for AMR with modification to suit the local perspective as needed.^13^

### Laboratory Methods

Blood cultures were analyzed with the BacTAlert 3D Microbial Detection system (bioMérieux, Marcy l’Etoile, France) for up to five days. Organisms were identified by Gram stain, growth on blood, chocolate, and MacConkey agar plates, and biochemical testing. Gram-positive organisms were distinguished using phenotypic methods, such as colony morphology, motility, hemolysis zones, bacitracin and optochin susceptibility, and catalase, coagulase, and PYR reactions. Gram-negative organisms were identified using the oxidase reaction and API 20E or API NE (bioMérieux, Marcy l’Étoile, France).^14^

Antimicrobial susceptibility testing (AST) was performed manually by disk diffusion, following Clinical Laboratory Standards Institute (CLSI) 2021 guidelines. Results were classified as susceptible, intermediate, or resistant. Extended-spectrum beta-lactamase (ESBL) production in Gram-negative isolates was tested with the disk diffusion clavulanate inhibition test.^14^ Blood culture results were communicated to clinical teams and attached to patient files.

### Audit Criteria and Standard

The Tanzanian STG was used as the agreed standard of comparison against the diagnosis in which ceftriaxone has been prescribed to treat bacterial infections in the surgical MNH-AICU. The National Essential Medicines List (NEMLIT) of the STG, 2021 presents essential medicines for which health facilities at all levels are requisite for the management of priority diseases.

### Data Management and Analysis of Bacterial Resistance to Ceftriaxone

Optimal antibiotic therapy (OAT) for ceftriaxone was defined as a well-established diagnosis and appropriate antibiotic use, following the performed AST results from different sources of cultures, such as blood, urine, wound swabs, pus swabs, tracheal aspirates or CSF. Then, the whole course of ceftriaxone therapy was evaluated in accordance with the current STG or with any other available internal/departmental/unit guidelines used as references at the surgical MNH-AICU. Any alteration of the first-line treatment was characterized as antibiotic reassessment.

A reduction in the number of prescribed antimicrobials or a narrower spectrum of activity of the medicine used as a second-line treatment was described as reassessment with antibiotic simplification.

The information extracted from the patients’ files was entered, coded and analyzed using SPSS, version 23, with results presented as frequencies, percentages and summary statistics for the studied events. To facilitate statistical data interpretation, continuous data were grouped and presented as the mean±SD and median with range, and discrete data were presented as percentages using frequency distribution tables/charts, and the results were compared to the standard guidelines.

### Ethical Considerations

Ethical approval was given by the Senate Research and Publications Committee of MUHAS (Ref. MUHAS-REC-03-2022-1043), and the Executive Director of MNH, gave permission to perform this study.

## RESULTS

### Sociodemographic Characteristics of the Study Group

During the 12 months of the study period, a total of 735 patients were admitted to the surgical MNH-AICU with different clinical diagnoses, but 4 of them were missing some of the information, including age and the number of procedures performed. As a result, 731 patients (99.5%) were included in the final analysis. The majority of the admitted patients were male 469, (64.2%), and only 275 (37.6%) had health insurance. The median age of the study group was 40 (28–57), for which the largest population was within the age category of 40–64 years, 320 (43.8%), followed by those of 18–39 years, 217 (29.7%), and finally those of 14–17 years, 25 (3.4%). The majority (716, 97.9%) were admitted as referrals, while 15 patients (2.1%) were self-referrals (Table 1).

**TABLE 1:** Baseline Sociodemographic Characteristics of the Septic and Nonseptic Patients Admitted to the AICU from January 2021 to December 2021 at MNH

Variable	STG Adherence N=26/731	No STG Adherence N=705/731
Gender		
Male	18 (69.20)	451 (64.00)
Female	8 (30.80)	254 (36.00)
Age	40 (28–57)	39 (28–56)
14–17	25 (3.40)	
18–39	217 (29.70)	
40–64	320 (43.80)	
>=65	169 (23.10)	
Insurance		
Insured	275 (37.60)	
None Insured	456 (62.40)	
Mode of Admission		
Self-referral	15 (2.10)	
Referral	716 (97.90)	

Note: STG=Standard Treatment Guideline.

### Baseline Clinical Characteristics of Patients Admitted to the AICU at MNH

Most of our patients were referred to the surgical MNH-AICU due to shock (108, 14.8%), low GCS (77, 10.5%) and postoperative (59, 8.1%). The most common specialties were medicine (526, 72.0%), followed by surgery (101, 13.8%).

A total of 279 (38.1%) patients had a history of antibiotic use four weeks prior to the surgical MNH-AICU admission, 12 (1.7%) did not use any antibiotics, and 440 (60.2%) had unknown information on the use of antibiotics. During their stay in the surgical MNH-AICU, out of those 731 patients, 286 (39.1%) died, while 445 (60.9%) were transferred back to their wards alive.

### Prevalence of sepsis among adult patients admitted to the surgical MNH-AICU

Among all patients admitted, 231 (31.60%) were found to have clinical sepsis, and 75 (32.50%) were microbiologically confirmed to have culture-positive sepsis.

### Patterns of Bacterial Pathogens Isolated Among Septic Patients Admitted to the Surgical MNH-AICU.

**FIGURE 1:**
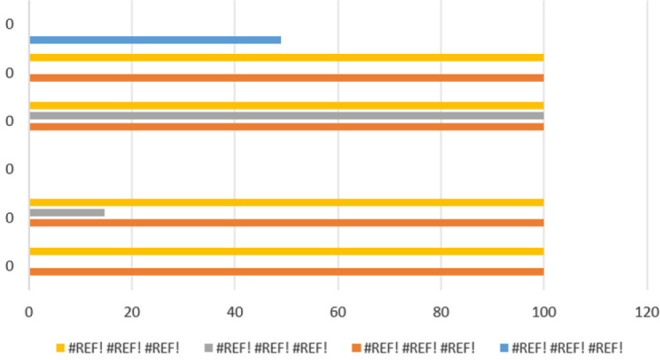
Patterns of Bacterial Pathogens Isolated with their Antibiotic Resistance Percentages.

Among all isolates, *K. pneumoniae* (34, 45%) was the most predominant bacterium, followed by *S. aureus* (19, 25%), *Acinetobacter* spp. (10, 13%), *Pseudomonas* spp. (7, 9%) and *E. coli* (5, 8%). *Klebsiella pneumoniae, Acinetobacter* spp. and *E. coli* were 100% resistant to ceftriaxone (CRO) and ampicillin (AMP), as shown in Table 2 below. Five isolates of *K. pneumoniae* were resistant to all tested antibiotics, including meropenem. Some of the bacteria were not tested for some specific antibiotics, as seen in the respective tables, due to the lack of antibiotics (Table 2 / Figure 1).

**TABLE 2:** Patterns of Bacterial Pathogens Isolated with their Antibiotic Resistance Percentages

Bacterial Isolate	n (%)	CRO (%)	MEM (%)	AMP (%)
*Pseudomonas spp*	7 (9.0)	100	0	-
*S.aureus*	19 (25.0)	100	0	-
*E.coli*	5 (8.0)	100	0	100
*K.pneumoniae*	29 (38.0)		14.7	100
	5 (7.0)	100	100	100
*Acinetobacter spp*	10 (13.0)	100	0	100
MDR (Total)	49 (65.30)			

Note: No resistance (0), Not tested (−), CRO=Ceftriaxone, MEM=Meropenem, AMP=Ampicillin.

### The use of ceftriaxone as the first-line antibacterial agentt and STG adherence

A total of 598 (81.8%) patients were prescribed ceftriaxone during their stay in the MNH-AICU. Among them, all patients who received ceftriaxone by adhering to the STG (4.30%), had the diagnosis of pneumonia with the differential diagnosis of COVID-19. The rest of the patients who received ceftriaxone, 572 (95.7%) were prescribed ceftriaxone without the STG adherence.

## DISCUSSION

The majority of the patients were male. The leading diagnosis was shock (14.8%) with a sepsis prevalence of 31.60%. The most common bacteria isolate was *K. pneumoniae* (34, 45%), showing 100% resistance to ceftriaxone. This may cause a sharp rise in mortalities. The spread of *K. pneumoniae* resistant strains in public hospitals is dangerous, especially where resources are limited.^15^

The 75 (32.50%) patients who had cultures performed represent less than half of all patients who were clinically diagnosed with sepsis. Some of the challenges that were put forward by the physicians in the surgical MNH-AICU were the absence/non-reliable supply of bottles for culture, as well as the logistics to collect bottles, since they had to be collected from the laboratory. Both of these issues have made things difficult and delayed starting empiric antibiotic coverage, bringing in the fear of increasing morbidity and mortality.^13,16^ We highly believe those factors contributed to the poor performance of cultures for all patients who happened to be candidates for cultures of different types.

We believe the high resistance to ceftriaxone (100%) is due to it being commonly prescribed as the first-line antibacterial agent in the surgical MNH-AICU, same as found in the study conducted in Ethiopia.^17^ Most patients had prior exposure to ceftriaxone at peripheral hospitals before referral to MNH. During the COVID-19 pandemic, ceftriaxone use increased as an antibacterial treatment, further raising resistance risks.^18^

We might have noticed a higher prevalence compared to other studies performed in other LMICs, which could be because other studies categorized sepsis into sepsis, severe sepsis, and septic shock, as reported in other studies,^19,20^ while in the surgical MNH-AICU, all septic patients were not categorized into specific categories of severity. The other reason could be misdiagnosis of sepsis, since in the present study, sepsis was diagnosed basing mostly on the physician's evaluation of the presenting clinical features and not supported by all recommended laboratory criteria. It has been shown that the diagnosis of sepsis is quite challenging, especially in regard to LMICs, where there are limited resources.^21,22^

In addition, other potential risks for hospital acquired sepsis such as poor adherence to aseptic measures by health workers in the surgical MNH-AICU team, as well as a lack of awareness of sepsis as a problem at our hospital, which could have contributed to the observed findings were not examined in our study.

At MNH, the majority of the patients come as referrals from the upcountry regions as well as within the Dar es Salaam region itself, including regional hospitals and health centers, for which there are different levels of skilled personnel.^13^ These may present different knowledge and hence different prescribing habits,^21^ hence the high resistance level toward ceftriaxone could be due to multiple factors.

Although not evaluated, some factors such as over prescription, patients not finishing the entire antibiotic course, poor infection control in health care settings, poor hygiene and sanitation,^23^ and the overuse of antibiotics in livestock and fish farming could be potential risk areas that warrant further research.^24^

Ceftriaxone has been demonstrated to be one of the most commonly used and prescribed antibiotics for many different types of diagnoses and conditions by many physicians. One study conducted at MNH medical wards,^1^ showed very high use of ceftriaxone. Similar findings on the high use of ceftriaxone were seen in other studies, as well as KCMC.^23^

In this study, we found that ceftriaxone was used in many other conditions, for which only 4.30% received ceftriaxone by the STG adherence, having pneumonia with the differential diagnosis of COVID-19. This may be due to fear from physicians as a result of the high mortality incurred in many COVID-19 patients worldwide,^25^ hence the precise and strict prescriptions as per guidelines requirements from many physicians.

Adherence to the STG could have been massively low outside of the COVID-19 population due to the fact that spreading of bacterial infections among patients, as well as health care providers, does not cause symptoms immediately compared to viral infections. That might lead to little concerns, less fear, hence negligence of all AMS measures as well as STG adherence.^26^

All of the physicians/clinicians involved in daily activities in the surgical MNH-AICU were very aware of the presence of STG that needs to be adhered for best practice. However, the STG was not available in specific locations in hard copies, and very few had the soft copies on their mobile phones. All of which made it difficult to access the STG and possibly contributed to the low adherences observed. Previous works revealed that readily availability of guidelines to all staff especially in soft copies on their smart mobile phones made it possible to access the guidelines at any time of the day and from any place as a reference to guide their management of all patients with either suspicion or confirmed COVID-19.^27–29^

We believe that putting in place the specific Antimicrobial Stewardship Program (ASP) team to work as the champions to pioneer the use of AMS in the surgical MNH-AICU would greatly improve the proper/correct use of ceftriaxone as well as other antibacterial agents. This could improve adherence to the STG for all prescribers in the country.^30,31^ Other interventions, such as persuasive (i.e. education, guidelines, reminders, regular audit and feedback) or restrictive measures like formulary restriction, compulsory order forms, expert approval, newer technologies,^32^ and automatic stop orders, could be incorporated in order to improve our evidence-based clinical practice to combat AMR.^30^

Our study based entirely on physicians’ diagnosis and treatment plan; hence, there is potential for bias as well as strengths, and therefore, there is a need to conduct more studies in the future to address these challenges.

## CONCLUSION

There was a significant proportion of sepsis due to MDR gram-negative bacteria, the majority of which were 100% resistant to ceftriaxone. The STG adherence with regard to the use of cefriaxone, was very low representing less than 5% of all prescription incidences. We recommend that ceftriaxone should no longer be used in the surgical MNH-AICU. There is an urgent need to implement the use of another drug or group as the first line for prophylaxis and treatment of bacterial infections in the surgical AICU at MNH.

Crucially, the establishment of dedicated ASP teams, along with rigorous education and strict adherence to defined guidelines, is of paramount importance to counteract and effectively reduce the observed antimicrobial resistance burden.

### Limitations

Our study did not include other microbes as the cause of sepsis, such as parasites, fungi and viruses.
